# miR-344-5p Modulates Cholesterol-Induced β-Cell Apoptosis and Dysfunction Through Regulating Caveolin-1 Expression

**DOI:** 10.3389/fendo.2021.695164

**Published:** 2021-07-28

**Authors:** Xulong Sun, Guangnian Ji, Pengzhou Li, Weizheng Li, Jun Li, Liyong Zhu

**Affiliations:** Department of General Surgery, The Third Xiangya Hospital, Central South University, Changsha, China

**Keywords:** β-cell, miR-344-5p, Caveolin-1, apoptosis, type 2 diabetes mellitus

## Abstract

Diabetes is a metabolic disorder induced by the modulation of insulin on glucose metabolism, and the dysfunction and decreased number of islets β-cells are the main causes of T2DM (type 2 diabetes mellitus). Among multiple factors that might participate in T2DM pathogenesis, the critical roles of miRNAs in T2DM and β-cell dysfunction have been reported. Through bioinformatics analyses and literature review, we found that miR-344 might play a role in the occurrence and progression of diabetes in rats. The expression levels of miR-344-5p were dramatically decreased within cholesterol-stimulated and palmitic acid (PA)-induced rats’ islet β-cells. In cholesterol-stimulated and PA-induced diabetic β-cell model, cholesterol-caused and PA-caused suppression on cell viability, increase in intracellular cholesterol level, decrease in GSIS, and increase in lip droplet deposition were dramatically attenuated *via* the overexpression of miR-344-5p, whereas aggravated *via* the inhibition of miR-344-5p. miR-344-5p also inhibited cholesterol-induced β-cell death *via* affecting the apoptotic caspase 3/Bax signaling. Insulin receptor downstream MPAK/ERK signaling was involved in the protection of miR-344-5p against cholesterol-induced pancreatic β-cell dysfunction. Moreover, miR-344-5p directly targeted Cav1; Cav1 silencing could partially reverse the functions of miR-344-5p inhibition upon cholesterol-induced β-cell dysfunction, β-cell apoptosis, the apoptotic caspase 3/Bax signaling, and insulin receptor downstream MPAK/ERK signaling. In conclusion, the miR-344-5p/Cav1 axis modulates cholesterol-induced β-cell apoptosis and dysfunction. The apoptotic caspase 3/Bax signaling and MAPK/ERK signaling might be involved.

## Introduction

Diabetes, a metabolic disorder induced by the modulation of insulin on glucose metabolism, is characterized by abnormal glucose homeostasis leading to elevated blood sugar and eventually resulting in damage to various organs of the body. The dysfunction and decreased number of islets β-cells are the main causes of T2DM (type 2 diabetes mellitus) (1).

Obesity (2, 3) and elevated plasma and islet cholesterol levels (4) represent an additional risk in T2DM development. With the occurrence of overnutrition and obesity, the level of free fatty acids in human plasma increased significantly. The level of visceral fat deposition and free fatty acid increase caused by obesity are the key factors leading to lipotoxicity on islets β-cells, leading to dysfunction and even failure of islet β-cells (5). Under the condition of massive obesity, a large number of free fatty acid inflows into the islets, increasing the secretion pressure, subsequently resulting in β-cell deterioration and even apoptosis (6). In the meantime, β-cells are functionally deteriorated, manifested by a decrease in GSIS (glucose-stimulated insulin secretion) (7–9). Protection against lipotoxicity on islets β-cells might be an effective strategy for treating T2DM (4).

MicroRNAs (miRNAs) constitute a class of small endogenous non-coding RNAs, which interact with the 3′-UTR (3′-untranslated region) of target mRNAs to inhibit post-transcriptional gene expression (10). miRNAs play a vital role in cell proliferation, cell cycle progression, cell apoptosis, cell differentiation, and many other crucial cell processes (11–14). Over the past years, the critical roles of miRNAs in T2DM and β-cell dysfunction have been reported (15–17). miRNAs not only regulate insulin secretion, islet development, islet β-cell differentiation, glucose metabolism, and lipid metabolism, but also contribute to the occurrence of diabetes and its complications (15–17). To identify miRNAs that might participate in islets β-cells apoptosis and dysfunctions, we downloaded and analyzed Gene Expression Omnibus (GEO) dataset GSE110234, which reported altered miRNA expression profiles in the streptozotocin-induced diabetic rats compared to normal Sprague Dawley (SD) rats. Differentially expressed miRNAs in dorsal root ganglia tissues between diabetic and normal rats based on GSE110234 were listed in **Table S1**, and it was found that miR-344-5p was significantly under-expressed in dorsal root ganglia tissues of diabetic SD rats (**Figures S1A, B**). Another study also indicated that miR-344 is downregulated in spontaneously diabetic Goto-Kakizaki rats (18). These previous findings imply that miR-344 may participate in the occurrence and progression of diabetes in rats; nevertheless, the specific role and the mechanism remain unclear.

Herein, we intend to determine the functions and the mechanism of the abnormally downregulated miR-344-5p in rat diabetes. We established cholesterol-induced lipotoxicity model in rats’ pancreatic islet β-cell, INS-1 cells, and evaluated the functions of miR-344-5p upon β-cell viability, intracellular cholesterol levels, GSIS (glucose-stimulated insulin secretion) by β-cell, and the cellular lipid deposition. Then, the effects of miR-344-5p on β-cell apoptosis, apoptotic caspase 3/Bax signaling, and insulin receptor downstream MAPK/ERK signaling were examined. Regarding the mechanism, we examined the downstream targets of miR-344-5p, and Caveolin-1 (Cav1) was identified. The dynamic effects of miR-344-5p and Cav1 on β-cell functions, apoptotic caspase 3/Bax signaling, and insulin receptor downstream MAPK/ERK signaling were determined.

## Materials and Methods

### Pancreatic Tissue Samples

Type 2 diabetes mellitus (T2DM) patients (68.17 ± 5.08 year; Female/Male: 3/3) were recruited from Third Xiangya Hospital. Exclusion criteria were patients with other acute or chronic complications, SLE, AMI, uncontrolled hypertension, pregnant, or other serious illness. Six diabetic pancreatic tissues were collected from patients with pancreatic cancer, bile duct cancer, or duodenal papillary tumor with a type II diabetes history for at least 1 year; six none-diabetes normal pancreatic tissues were collected from pancreatic cancer, bile duct cancer, or duodenal papillary tumor patients (59.17 ± 6.49 year; Female/Male: 1/5) without diabetes. All sample tissues were frozen in liquid nitrogen and then stored at −80°C. All human specimens were obtained with the approval by the ethics committee of Third Xiangya Hospital, and all samples were supplied by patients who provided informed consent.

### Cell Line and Treatment

Rat β-cell line, INS-1, was obtained from the National Infrastructure of Cell Line Resource (Beijing, China) and cultured in RPMI-1640 (Invitrogen, Carlsbad, CA, USA) supplemented with L-glutamine, 10% FBS (Invitrogen), HEPES (10 mmol/L; Gibco, Waltham, MA, USA), sodium pyruvate (1 mmol/L; Sigma-Aldrich, St. Louis, MO, USA), and β-mercaptoethanol (50 μmol/L; Sigma). Cells were incubated at 37°C in 5% CO_2_. All experiments were performed using INS-1 cells between passage numbers from 4 to 20.

For high-fat induction, the water-soluble cholesterol (Cat. C4951-30MG, Sigma-Aldrich), which contained 47 mg of cholesterol/g solid, was dissolved in RPMI-1640 culture medium to reach the final cholesterol concentrations of 0, 2.5, 5, and 10 mM. INS-1 cells were treated with 0, 2.5, 5, or 10 mM cholesterol for 6, 12, or 24 h for high-fat induction following the methods described previously (19, 20). The final cholesterol concentrations were ascertained by measuring the levels of cell viability.

Palmitate acid (PA), the most common saturated free fatty acid, can lead to lipotoxicity and apoptosis when overloaded in pancreatic β-cell (21). PA (Sigma-Aldrich) was conjugated with fatty acid–free bovine serum albumin (BSA) before addition to cell culture. PA was dissolved in 99% ethanol and then mixed with 10% BSA in serum-free RPMI-1640 to make a 0.5 mM PA solution and used to treat cells for 24 h. An empty vehicle control of ethanol and fatty acid–free BSA without PA was used as a negative control (untreated) (22, 23).

### Cell Transfection

miR-344-5p overexpression or inhibition in INS-1 cells were achieved by transfecting miR-344-5p mimics (mimic NC transfected as a negative control) or miR-344-5p inhibitor (inhibitor NC transfected as a negative control). Caveolin-1 (Cav1) silencing was achieved in INS-1 cells by transfecting small interfering RNA targeting Cav1 (si-Cav1; si-NC transfected as a negative control). The sequence of Cav1 siRNA, miR-344-5p mimics, and miR-344-5p inhibitor are listed in **Table S2**. All the transfection plasmids were synthesized and obtained from Genetop (Changsha, China). All the transfections were performed using Lipofectamine^®^ 3000 reagent (Thermo Fisher Scientific, Waltham, MA, USA).

### MTT Assay Detecting Cell Viability

INS-1 cells were transfected or non-transfected and seeded into 96-well plates for 24 h at 37°C. After incubating with 10 µl of MTT (5 mg/ml; Sigma-Aldrich) for 4 h at 37°C, 150 µl of dimethyl sulfoxide (Sigma-Aldrich) was added to each well, and the plates were kept in dark for 20 min at room temperature. At the end of the incubation, the absorbance value was determined at 490 nm, and the cell viability was calculated, taking non-transfected cell viability as 100%.

### Flow Cytometry Detecting Cell Apoptosis

An annexin V-fluorescein isothiocyanate (FITC)/propidium iodide (PI) apoptosis detection kit (BD, Shanghai, China) was used to detect INS-1 cell apoptosis. Cells were centrifugated at 225 × g for 10 min, washed with phosphate-buffered saline, and resuspended in binding buffer (300 µl). Then, cells were incubated with 5 µl of annexin V-FITC solution for 15 min in dark and added with 5 µl PI. A flow cytometer (BD) was used to analyze cell apoptosis.

### Immunoblotting

For detecting the protein levels of Cav1, Cdc42, VAMP2, Bcl-2, Bax, cleaved-caspase 3, β-actin, p-AKT, AKT, p-MAPK, MAPK, p-ERK, ERK, β-actin and GAPDH, the total protein was extracted using a ProteoPrep^®^ total extraction sample kit (Sigma-Aldrich), resolved on 10% sodium dodecyl sulfate (SDS)-polyacrylamide gels, and electrophoretically transferred onto polyvinylidene fluoride (PVDF) membranes. Membranes were first incubated with 5% non-fat dry milk in Tris-buffered saline Tween (TBST) for 2 h for blocking non-specific bindings. Then, membranes were incubated with appropriate primary antibodies, respectively, at 4°C overnight followed by another incubation with appropriate secondary antibodies for 2 h at room temperature. Used primary antibodies are Bcl-2 (1:1,000 dilution, 12789-1-AP, Proteintech, Wuhan, China), Bax (1:1,000 dilution, 50599-2-Ig, Proteintech), cleaved-caspase 3 (1:50 dilution, ab2302, Abcam, Cambridge, MA, USA), p-AKT (1:500 dilution, Y011054; ABM, Richmond, BC, USA), AKT (1:500 dilution, Y409094, ABM), MAPK (1:1,000 dilution, 33-1300; Invitrogen), p-MAPK (1:1,000 dilution, 44-684G, Invitrogen), p-ERK (1:1,000 dilution, sc-81492; Santa Cruz, Dallas, TX, USA), ERK (1:1,000 dilution, 67170-1-Ig, Proteintech), β-actin (internal reference; 60008-1-Ig, Proteintech), and GAPDH (internal reference; ab8245; Abcam). After washing three times for 10 min each time in Tris-buffered saline and Tween 20, membranes were incubated with HRP-labeled goat anti-rabbit IgG (1:500 dilution, Santa Cruz) at room temperature for 1 h. The immunoreactive proteins were visualized and examined using an enhanced chemiluminescence reagent (ECL; BeyoECL Star Kit, Beyotime, Shanghai, China), and the relative protein levels were calculated, normalizing to β-actin or GAPDH.

### Polymerase Chain Reaction (PCR)–Based Analysis

Total RNA was extracted, processed, and examined for the expression of target genes according to a method described previously (24). The oligo-dT or stem-loop reverse transcriptase primers were used to obtain cDNA. The expression levels of target miRNAs or mRNAs were detected by SYBR green PCR Master Mix (Qiagen, Hilden, Germany) taking β-actin (for mRNAs) or U6 (for miRNAs) as an endogenous control. The data were processed using a 2^−ΔΔCT^ method. The primer sequence was listed in **Table S3**.

### Intracellular Cholesterol Level

The cellular contents of cholesterol in INS-1 cells were measured using cholesterol assay kits (ab133116; Abcam) according to the manufacturers’ protocol.

### Glucose-Stimulated Insulin Secretion (GSIS) Measurements

Cells were washed with PBS and equilibrated in Kerbs-Ringer bicarbonate buffer (KRBB, pH 7.4) containing 140 mM NaCl, 1.5 mM CaCl_2_, 0.5 mM KH_2_PO_4_, 3.6 mM KCl, 0.5 mM MgSO_4_, 2 mM NaHCO_3_, 10 mM HEPES, and 0.1% BSA at 37°C for 30 min. The buffer was removed and replaced with fresh KRBB containing 2.8 or 16.7 mmol/L glucose for 1 h. Supernatants were collected, and the insulin concentration was measured using an insulin ELISA kit after appropriate dilution. Total protein was extracted with RIPA lysis buffer supplemented with 1 mM phenylmethyl sulfonylfluoride, and the protein concentration was determined using a BCA protein assay kit. The levels of insulin secretion were normalized against the respective protein content. Insulin secretion following stimulation with 2.8 and 16.7 mmol/L glucose was defined as basal insulin secretion and glucose-stimulated insulin secretion, respectively.

### Oil Red O Staining Detecting Lipid Deposition

After treatment and/or transfection, INS-1 cells were fixed in paraformaldehyde (4%), stained with oil red O (0.5%; Santa Cruz, Dallas, TX, USA), and observed for images under an inverted light microscope.

### Dual-Luciferase Reporter Assay

For validating the binding between miR-344-5p and Cav1 3’-UTR, dual-luciferase reporter assay was performed. The 3’-UTR of Cav1 was amplified by PCR using genomic DNA of the 293T cell line and cloned downstream of the Renilla luciferase open reading frame in the Renilla psiCHECK2 vector (Promega, Madison, WI, USA) using XhoI and NotI restriction sites. Mutations were introduced to the seed region of the miR-344-5p binding site in the Cav1 3’-UTR, and the construct was named mut-Cav1 3’-UTR. Next, 293T cells were seeded in 96-well plates and co-transfected with agomir-532-3p/antagomir-532-3p together with psiCHECK-2 reporter vectors (wt-/mut-Cav1 3’-UTR). Forty-eight hours after transfection, luciferase activity was measured using the Dual-Luciferase Reporter Assay System (Promega) according to the manufacturer’s instructions. Values were double normalized to firefly luciferase activity and to cells transfected with empty psiCHECK-2 control vectors.

### Statistical Analysis

All the experiments were repeated for at least three times. Data from at least three independent experiments were processed using SPSS17.0 (IBM, Armonk, NY, USA) and then presented as the mean ± S.D. A Student *t-*test was used for statistical comparison between means where applicable. Differences among more than two groups in the above assays were estimated using one-way ANOVA analysis follow Tukey *post-hoc* test. A *P* value of <0.05 is considered significantly different.

## Results

### Cholesterol-Induced and Palmitic Acid (PA)–Induced Lipotoxicity on Rat Pancreatic β-cell INS-1 Cells

**Figure 1:**
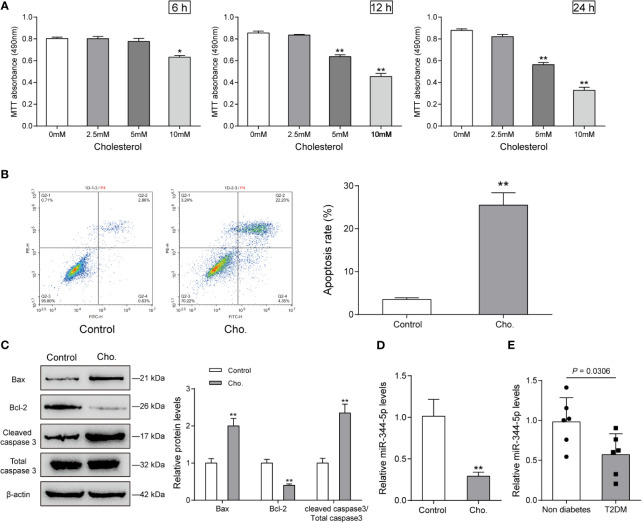
Cholesterol-induced cytotoxicity on rat pancreatic β-cell INS-1 cells. **(A)** INS-1 cells were treated with 0, 2.5, 5, or 10 mM cholesterol for 6, 12, or 24 h for high-fat induction and examined for cell viability by MTT assay. Then, INS-1 cells were treated with 5 mM cholesterol for 12 h and examined for the cell apoptosis by Flow cytometry assay **(B)**; the protein levels of Bcl-2, Bax, and cleaved-caspase 3 by Immunoblotting, β-actin was applied as an endogenous control **(C)**; the expression levels of miR-344-5p by real-time PCR **(D)**. **(E)** The expression level of miR-344-5p in pancreatic tissues of patients with type 2-diabetes (n=6) and none-diabetes patients (n=6) was detected by real-time PCR. *P < 0.05, **P < 0.01.

As we have mentioned, miR-344-5p expression was significantly downregulated in diabetic rats’ islets (**Figures S1A, B** and **Table S1**); to investigate the specific functions of miR-344-5p upon lipotoxicity-induced pancreatic β-cell dysfunction, we established cholesterol-induced and PA-induced lipotoxicity model in rats’ pancreatic β-cell INS-1 cell line. We treated INS-1 cell line with 0, 2.5, 5, or 10 mM cholesterol for 6, 12, or 24 h or 0.5 mM PA for 24 h for lipotoxicity induction; as revealed by MTT assay, 10 mM cholesterol treating 6, 12, or 24 h, and 5 mM cholesterol treating 12 h or 24 h significantly inhibited the cell viability of INS-1 cell (**Figure 1A**). Cell viability was decreased with PA treatment in INS-1 cell (**Figure S2A**). Then, we chose 5 mM cholesterol treatment for 12 h for further experiments because it is the minimal effective concentration inhibiting INS-1 cell viability. As revealed by Flow cytometry assay, 5 mM cholesterol treatment for 12 h or 0.5 mM PA treatment for 24 h significantly promoted cell apoptosis (**Figure 1B** and **Figure S2B**). Consistently, cholesterol or PA treatment significantly upregulated Bax and cleaved-caspase 3 protein contents, but downregulated Bcl-2 protein (**Figure 1C** and **Figure S2C**). These data indicate that 5 mM cholesterol treatment for 12 h or 0.5 mM PA treatment for 24 h successfully induces lipotoxicity on INS-1 cells. Upon cholesterol-induced or PA-induced lipotoxicity, miR-344-5p expression showed to be significantly decreased (**Figure 1D** and **Figure S2D**). Besides, when compared with the non-diabetic control subjects (n = 6), the patients with type 2 diabetes (n = 6) exhibited lower pancreatic tissue miR-344-5p expression level (**Figure 1E**).

### Effects of miR-344-5p Upon Cholesterol-Induced and PA-Induced β-Cell Dysfunction

**Figure 2:**
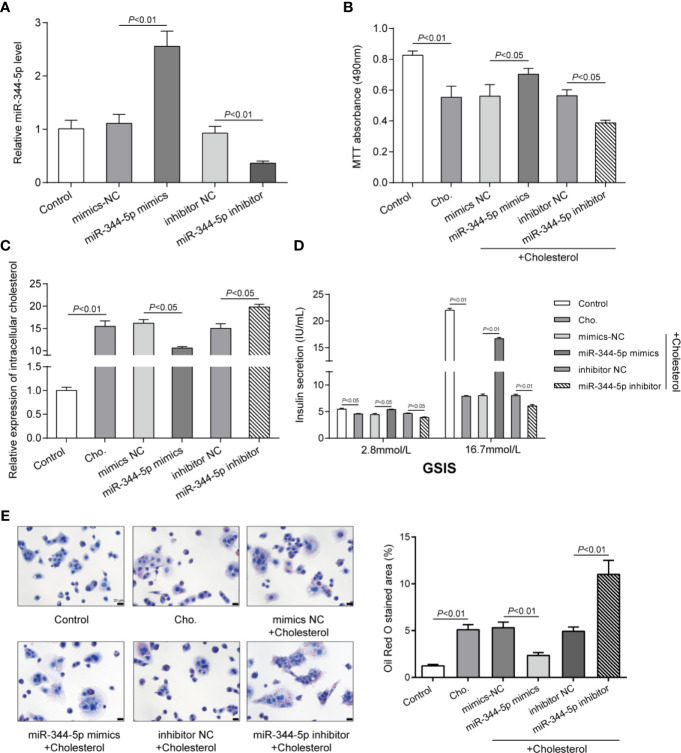
Effects of miR-344-5p on cholesterol-induced β-cell dysfunction. **(A)** miR-344-5p overexpression or inhibition was achieved in INS-1 cells by transfecting miR-344-5p mimic or inhibitor. The transfection efficiency was verified using real-time PCR 48 h after transfection. Then, pancreatic β-cell line, INS-1, was transfected with miR-344-5p mimic or inhibitor for 48 h, treated with 5 mM cholesterol for 12 h, and examined for cell viability by MTT assay **(B)**; intracellular cholesterol levels by cholesterol assay kits **(C)**; glucose-stimulated insulin secretion (GSIS) by an insulin ELISA kit **(D)**; the fat droplets deposition by Oil Red O staining **(E)**.

To detect the specific functions of miR-344-5p, we achieved miR-344-5p overexpression or inhibition in INS-1 cells by transfecting miR-344-5p mimic or inhibitor. We performed real-time PCR to verify the transfection efficiency 48 h after transfection (**Figure 2A**). Then, we transfected INS-1 cells with miR-344-5p mimic/inhibitor for 48 h, treated them with 5 mM cholesterol for 12 h or 0.5 mM PA treatment for 24 h, and examined for β-cell functions. Cholesterol or PA treatment significantly inhibited cell viability, which was partially reversed by miR-344-5p overexpression but further inhibited by miR-344-5p inhibition (**Figure 2B** and **Figure S2E**). Consistently, cholesterol or PA treatment significantly increased intracellular cholesterol levels, which were significantly reduced by miR-344-5p overexpression but further increased by 344-5p inhibition (**Figure 2C** and **Figure S2F**). Next, the GSIS by β-cells was examined using an insulin ELISA kit; as shown in **Figure 2D** and **Figure S2G**, cholesterol or PA treatment significantly suppressed the GSIS by β-cells, which showed to be dramatically reversed *via* the overexpression of miR-344-5p but further inhibited *via* the inhibition of miR-344-5p. As for the fat droplets deposition, cholesterol treatment significantly increased the fat droplets deposition, which was partially reduced by miR-344-5p overexpression but further increased by 344-5p inhibition (**Figure 2E**). These data indicate that cholesterol or PA treatment suppresses β-cell viability, suppresses GSIS by β-cells, and increases the fat droplets deposition; cholesterol or PA treatment–induced β-cell dysfunction could be attenuated by miR-344-5p overexpression but further aggravated by 344-5p inhibition.

### miR-344-5p Attenuates Cholesterol-Induced β-Cell Apoptosis and the MAPK/ERK Signaling Deregulation

**Figure 3:**
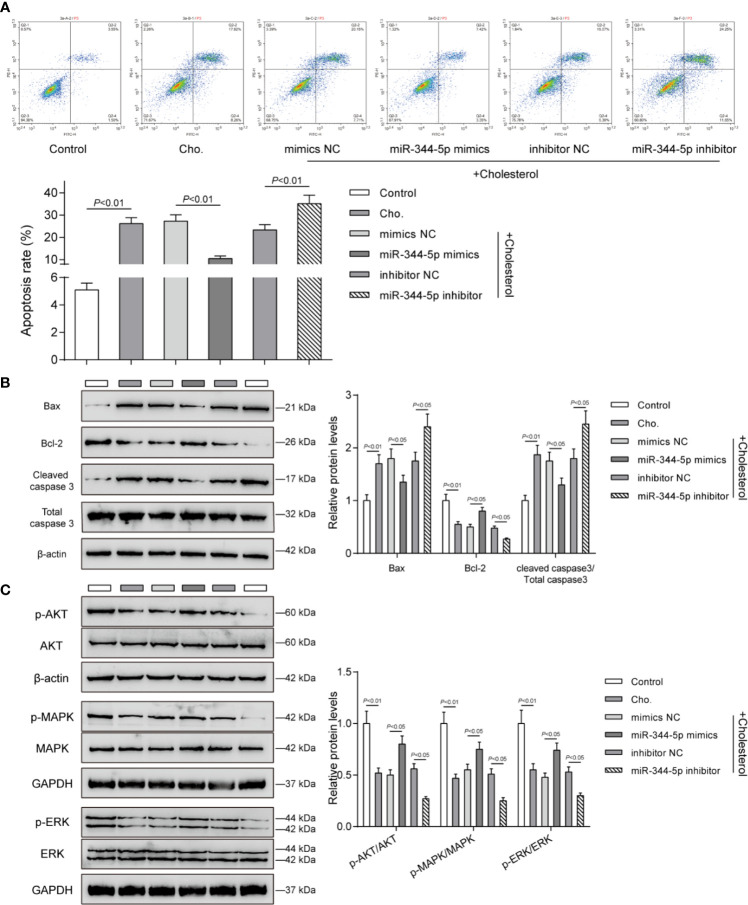
miR-344-5p attenuates cholesterol-induced β-cell apoptosis and the MAPK/ERK signaling deregulation. Pancreatic β-cell line, INS-1, was transfected with miR-344-5p mimic or inhibitor for 48 h, treated with 5 mM cholesterol for 12 h, and examined for cell apoptosis by Flow cytometry assay **(A)**; the protein levels of Bax, Bcl-2, and cleaved-caspase 3 by Immunoblotting **(B)**; the protein levels of p-AKT, AKT, p-MAPK, MAPK, p-ERK, and EKR by Immunoblotting **(C)**. β-actin and GAPDH were applied as an endogenous control.

Since cholesterol treatment significantly affects pro-apoptotic Bax, anti-apoptotic Bcl-2, and pro-apoptotic cleaved-caspase 3, next, the effects of miR-344-5p upon β-cell apoptosis and these apoptosis regulators were determined. INS-1 cells were transfected and treated accordingly. Cholesterol treatment significantly promoted β-cell apoptosis, which was inhibited *via* the overexpression of miR-344-5p while further promoted *via* the inhibition of miR-344-5p (**Figure 3A**). Consistently, cholesterol treatment–induced increases in pro-apoptotic Bax and cleaved-caspase 3 and decrease in anti-apoptotic Bcl-2 were partially reversed by miR-344-5p overexpression but further enhanced by miR-344-5p inhibition (**Figure 3B**).

The components of MAPK/ERK signaling pathway have been considered to be the regulators of cellular insulin response. The decrease in gene expression of insulin-like receptor after persistent inhibition of MAPK/ERK signaling pathway could lead to insulin resistance (25). MAPK/ERK inhibition sensitized β-cell line MIN6 to stress-mediated cell death and lipotoxicity (26). Next, we investigated whether miR-344-5p could affect the MAPK/ERK signaling pathway. Consistent with the previous studies, cholesterol treatment significantly decreased the ratio of p-AKT/AKT, p-MAPK/MAPK, and p-ERK/EKR; miR-344-5p overexpression partially reversed, whereas miR-344-5p inhibition further enhanced cholesterol treatment–induced changes in these factors (**Figure 3C**).

### miR-344-5p Directly Binds to Caveolin-1 (Cav1)

**Figure 4:**
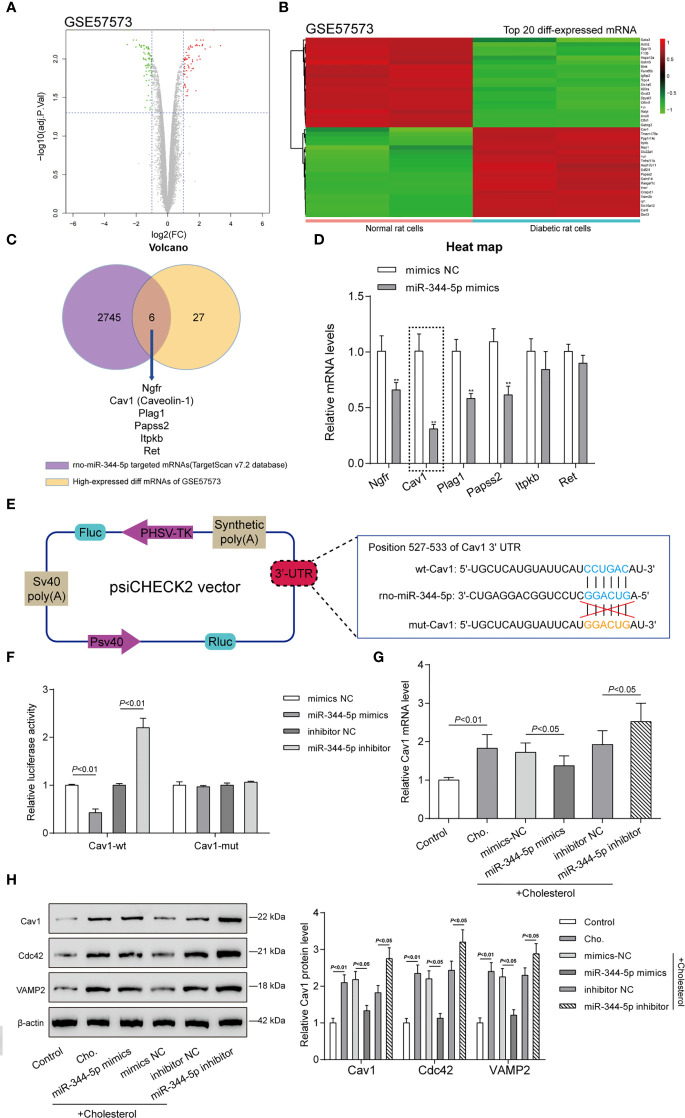
Caveolin-1 (Cav1) is a direct downstream target of miR-344-5p. **(A)** Volcano plots of abnormally upregulated and downregulated mRNAs between normal and human islet amyloid polypeptide (hIAPP)-treated INS-1E cells (type II diabetic cell model) based on GSE57573. Green plots represent 37 downregulated mRNAs, and red plots represent 33 upregulated mRNAs. **(B)** Hierarchical clustering heatmap showing the top 20 differentially expressed mRNAs between normal and hIAPP-treated INS-1E cells based on GSE57573. **(C)** TargetScan 7.2 was used to predict miR-344-5p target genes, and 2,751 mRNAs were obtained; these predicted target genes and 33 upregulated mRNAs in GSE57573 intersected at Ngfr, Cav1 (Caveolin-1), Plag1, Papss2, Itpkb, and Ret. Through literature review, Cav1 was chosen for further study. **(D)** INS-1 cells were transfected with miR-344-5p mimic and examined for these six genes’ expression levels by real-time PCR. **P < 0.01, **P < 0.001 compared to mimics NC group. **(E)** Diagram depicting the 3’-UTR reporter constructs. The Cav1 3′UTR fragments of rat were inserted into the psiCHECK-2 vector downstream of the Renilla luciferase. **(F)** Dual-luciferase reporter assay was performed to verify the predicted binding between miR-344-5p and Cav1. **(G, H)** INS-1 cells were transfected with miR-344-5p mimic or inhibitor for 48 h, treated with 5 mM cholesterol for 12 h, and examined for Cav1 mRNA **(G)** and Cav1, VAMP2, and Cdc42 protein **(H)** expression levels by real-time PCR and immunoblotting.

miRNAs interact with the 3′-UTR (3′-untranslated region) of target mRNAs to inhibit post-transcriptional gene expression (10). To investigate the mechanism underlying miR-344-5p protective effects against cholesterol-induced lipotoxicity on β-cells, GEO dataset GSE57573 was downloaded and analyzed to identify differentially expressed mRNAs in normal and human islet amyloid polypeptide (hIAPP)–treated INS-1E cells (type II diabetic cell model). As shown in **Figure 4A**, a total of 37 mRNAs were downregulated (green plots) and 33 were upregulated (red plots) in hIAPP-treated INS-1E cells. Hierarchical clustering heatmap showed the top 20 differentially expressed (20 upregulated and 20 downregulated) mRNAs between normal and hIAPP-treated INS-1E cells based on GSE57573 (**Figure 4B** and **Table S4**). Then, TargetScan 7.2 was used to predict miR-344-5p target genes, and 2,751 mRNAs were obtained; these predicted target genes and 33 upregulated mRNAs in GSE57573 intersected at Ngfr, Cav1 (Caveolin-1), Plag1, Papss2, Itpkb, and Ret (**Figure 4C**). Then, INS-1 cells were transfected with miR-344-5p mimic and examined for these six genes’ expression levels (**Figure 4D**). miR-344-5p mimic observably inhibited Ngfr, Cav1, Plag1, and Papss2 expression levels, and among them the Cav1 expression level was lowest. Besides, Cav1 has exerted its effect on insulin signaling and lipid metabolism within the liver, adipose tissues, and skeletal muscles to influence diabetes development (27). Thus, we chose Cav1 for further experiments.

Secondly, we verified the predicted binding between miR-344-5p and Cav1 by performing the dual-luciferase reporter assay. Based on M&M section, we constructed two different types of Cav1 luciferase reporter plasmids, wild-type and mutant-type, namely, wt-Cav1 and mut-Cav1 (**Figure 4E**). Then we co-transfected these plasmids in 293T cells with miR-344-5p mimics/inhibitor and examined for the luciferase activity. **Figure 4F** showed that miR-344-5p overexpression suppressed, whereas miR-344-5p inhibition promoted wt-Cav1 luciferase activity; when co-transfected with mut-Cav1, miR-344-5p overexpression or inhibition failed to alter the luciferase activity. In summary, miR-344-5p targets Cav1 at its 3′-UTR.

Cav1 interacts with Cdc42 and VAMP2 in β-cells (28). VAMP2 and Cdc42 localize together at the plasma membrane and on insulin secretory granules in β-cells and play vital functions in insulin secretion and development of diabetes (29). Hence, VAMP2 and Cdc42 were used as the positive control of Cav1 in the study. INS-1 cells were transfected with miR-344-5p mimic/inhibitor for 48 h, treated with 5 mM cholesterol for 12 h, and examined for Cav1 mRNA and protein and VAMP2 and Cdc42 protein expression levels. As shown in **Figure 4G**, cholesterol significantly induced the Cav1 mRNA expression; miR-344-5p overexpression downregulated, whereas miR-344-5p inhibition further upregulated cholesterol-induced Cav1 mRNA expression. As revealed in **Figure 4H**, cholesterol markedly promoted Cav1, Cdc42, and VAMP2 protein expression; overexpression of miR-344-5p downregulated, whereas inhibition of miR-344-5p further upregulated cholesterol-induced Cav1, Cdc42, and VAMP2 protein expression. Considering these findings, we speculate that miR-344-5p might exert its protective effects against cholesterol-induced lipotoxicity through targeting Cav1.

### miR-344-5p Protects Against Cholesterol-Induced β-Cell Dysfunction Through Cav1

**Figure 5:**
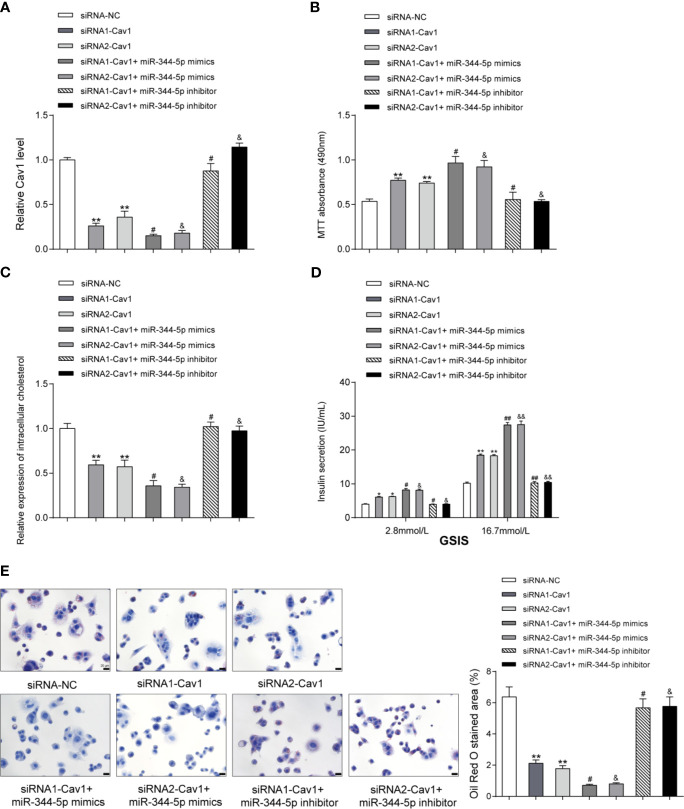
miR-344-5p protects against cholesterol-induced β-cell dysfunction through Cav1. **(A)** Cav1 silencing was achieved in INS-1 cells by transfecting small interfering RNA targeting Cav1 (siRNA1-Cav1 or siRNA2-Cav1); si-NC was transfected as a negative control. The transfection efficiency was verified using real-time PCR 48 h after transfection. Then, INS-1 cells were divided into seven groups: siRNA-NC group, siRNA1-Cav1 group, siRNA2-Cav1 group, siRNA1-Cav1+ miR-344-5p mimics, siRNA2-Cav1+ miR-344-5p mimics, siRNA1-Cav1+ miR-344-5p inhibitor, and siRNA2-Cav1+ miR-344-5p inhibitor. INS-1 cells in each group were transfected accordingly, treated with 5 mM cholesterol for 12 h, and examined for the expression of Cav1 by real-time PCR. **(B)** The cell viability was determined by MTT assay. **(C)** The intracellular cholesterol levels were examined by cholesterol assay kits. **(D)** The GSIS was determined by an insulin ELISA kit; **(E)** the fat droplets deposition was determined by Oil Red O staining. **P < 0.01 compared to siRNA-NC; ^#^ *P* < 0.05 compared to siRNA1-Cav1 group; ^&^ *P* < 0.05 compared to siRNA2-Cav1 group. *p < 0.05, ^##^p < 0.01, ^&&^p < 0.01.

As a further confirmation, next, the study investigates the specific functions of Cav1 upon cholesterol-induced lipotoxicity on β-cells. Cav1 silencing was achieved in INS-1 cells by transfecting small interfering RNA targeting Cav1 (siRNA1-Cav1 or siRNA2-Cav1); si-NC was transfected as a negative control. We performed the real-time PCR to verify the transfection efficiency 48 h after transfection (**Figure 5A**). Then, INS-1 cells were divided into seven groups: siRNA-NC group, siRNA1-Cav1 group, siRNA2-Cav1 group, siRNA1-Cav1+ miR-344-5p mimics, siRNA2-Cav1+ miR-344-5p mimics, siRNA1-Cav1+ miR-344-5p inhibitor, and siRNA2-Cav1+ miR-344-5p inhibitor. INS-1 cells in each group were transfected accordingly, treated with 5 mM cholesterol for 12 h, and examined for the expression of Cav1. As shown in **Figure 5A**, siRNA1-Cav1 or siRNA2-Cav1 transfection significantly downregulated and overexpression of miR-344-5p further inhibited Cav1 expression; the promotive effects of miR-344-5p inhibition on Cav1 expression were significantly reversed by siRNA1-Cav1 or siRNA2-Cav1.

Regarding the cellular functions, under cholesterol stimulation, the cell viability was significantly promoted by siRNA1-Cav1 or siRNA2-Cav1 and was further promoted by miR-344-5p mimics, but was inhibited by miR-344-5p inhibitor; the suppressive effects of miR-344-5p inhibitor on β-cell viability were significantly reversed by siRNA1-Cav1 or siRNA2-Cav1 (**Figure 5B**). Consistently, under cholesterol stimulation, the intracellular cholesterol levels were reduced by siRNA1-Cav1 or siRNA2-Cav1 and were further reduced by miR-344-5p mimics, but were increased by miR-344-5p inhibitor; the promotive effects of miR-344-5p inhibitor on intracellular cholesterol levels were significantly reversed by siRNA1-Cav1 or siRNA2-Cav1 (**Figure 5C**). Under cholesterol stimulation, siRNA1-Cav1 or siRNA2-Cav1 promoted, and miR-344-5p mimics further promoted, whereas miR-344-5p inhibitor suppressed the GSIS by β-cells; the suppressive effects of miR-344-5p inhibitor on GSIS by β-cells were significantly reversed by siRNA1-Cav1 or siRNA2-Cav1 (**Figure 5D**). Under cholesterol stimulation, siRNA1-Cav1 or siRNA2-Cav1 significantly reduced, and miR-344-5p mimics further reduced, whereas miR-344-5p inhibitor increased the fat droplets deposition; the promotive effects of miR-344-5p inhibitor on the fat droplets deposition were significantly reversed by siRNA1-Cav1 or siRNA2-Cav1 (**Figure 5E**).

### The miR-344-5p/Cav1 Axis Affects Cholesterol-Induced β-Cell Dysfunction Through the Apoptosis and MAPK/ERK Signaling

**Figure 6:**
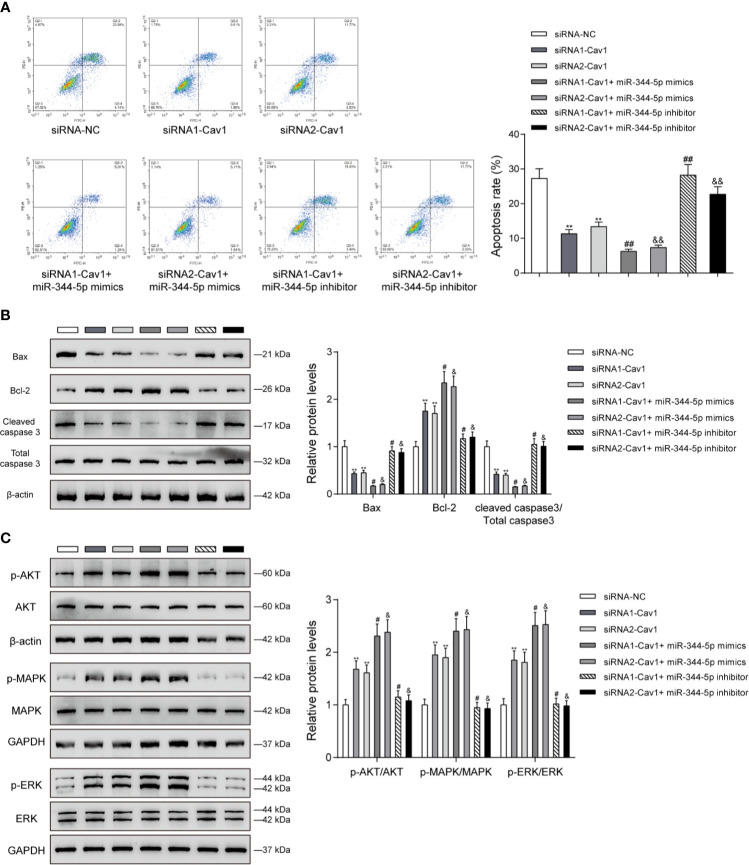
The miR-344-5p/Cav1 axis affects cholesterol-induced β-cell dysfunction through the apoptosis and MAPK/ERK signaling. INS-1 cells were divided into seven groups as described above and were transfected accordingly, treated with 5 mM cholesterol for 12 h, and examined for cell apoptosis by Flow cytometry assay **(A)**; the protein levels of Bax, Bcl-2, and cleaved-caspase 3 by immunoblotting **(B)**; the protein levels of p-AKT, AKT, p-MAPK, MAPK, p-ERK, and EKR by immunoblotting **(C)**. β-actin and GAPDH were applied as an endogenous control. **P < 0.01 compared to siRNA-NC; ^#^ *P* < 0.05 compared to siRNA1-Cav1 group; ^&^ *P* < 0.05 compared to siRNA2-Cav1 group. ^##^p < 0.01, ^&&^p < 0.01.

Since miR-344-5p affects the apoptosis and MAPK/ERK signaling under cholesterol stimulation, next, the study examined the dynamic effects of the miR-344-5p/Cav1 axis upon β-cell apoptosis and related signaling factors. INS-1 cells were divided into seven groups as mentioned above and treated with 5 mM cholesterol for 12 h accordingly. Under cholesterol stimulation, siRNA1-Cav1 or siRNA2-Cav1 significantly inhibited, and miR-344-5p mimics further inhibited, whereas miR-344-5p inhibitor promoted the cell apoptosis; the promotive effects of miR-344-5p inhibitor on cell apoptosis were significantly reversed by siRNA1-Cav1 or siRNA2-Cav1 (**Figure 6A**). Consistently, under cholesterol stimulation, siRNA1-Cav1 or siRNA2-Cav1 or miR-344-5p mimics downregulated Bax and cleaved-caspase 3 protein contents and upregulated Bcl-2 protein, whereas miR-344-5p inhibitor exerted opposite effects; the effects of miR-344-5p inhibitor on these three proteins were significantly reversed by siRNA1-Cav1 or siRNA2-Cav1 (**Figure 6B**). Finally, under cholesterol stimulation, siRNA1-Cav1 or siRNA2-Cav1 significantly increased, and miR-344-5p mimics further increased, whereas miR-344-5p inhibitor decreased the ratio of p-AKT/AKT, p-MAPK/MAPK, and p-ERK/ERK; the suppressive effects of miR-344-5p inhibitor on AKT, MAPK, and ERK phosphorylation were significantly reversed by siRNA1-Cav1 or siRNA2-Cav1 (**Figure 6C**).

## Discussion

Herein, the study confirmed that the expression levels of miR-344-5p were dramatically decreased within diabetic rats and cholesterol-stimulated rats’ islet β-cells. In cholesterol-stimulated diabetic β-cell model, cholesterol-caused suppression on cell viability, increase in intracellular cholesterol level, decrease in GSIS, and increase in lip droplet deposition were dramatically attenuated *via* the overexpression of miR-344-5p, whereas aggravated *via* the inhibition of miR-344-5p. miR-344-5p also inhibited cholesterol-induced β-cell death *via* affecting the apoptotic caspase 3/Bax signaling. Insulin receptor downstream MPAK/ERK signaling was involved in the protection of miR-344-5p against cholesterol-induced pancreatic β-cell dysfunction. Moreover, miR-344-5p directly targeted Cav1; Cav1 silencing could partially reverse the functions of miR-344-5p inhibition upon cholesterol-induced β-cell dysfunction, β-cell apoptosis, the apoptotic caspase 3/Bax signaling, and insulin receptor downstream MPAK/ERK signaling.

miRNA deregulation in diabetes has been reported previously. Kong et al. (30) reported that miR-9, miR-29a, miR-30d, miR-34a, miR-124a, miR146a, and miR-375 could be involved in insulin regulation and T2DM development. Besides, within pre-T2DM stage, the expression levels of these miRNAs are not altered significantly, undermining their effectiveness as disease-specific biomarkers. Another report by Karolina et al. (31) indicated that miR-150, miR-192, miR-27a, miR-320a, and miR-375 showed to be increased within T2DM. Herein, online dataset GSE110234 indicated the abnormal downregulation of miR-344-5p in diabetic rats. In cholesterol-stimulated rats’ islet β-cells, miR-344-5p expression also showed to be remarkably decreased. Moreover, miR-344 has been reported to be downregulated within pancreas islets from the spontaneously diabetic Goto-Kakizaki (GK) rats (18). In summary, miR-344-5p might contribute to β-cell dysfunction pathogenesis during T2DM.

T2DM development is implicated in gradual degradation of β-cell function without a significant change in insulin sensitivity (32). Normally islet β-cells respond to insulin resistance by increased secretion through the processes of compensation. These include an expansion of β-cell mass, increased insulin biosynthesis, and enhanced nutrient secretion coupling processes with increased sensitivity to glucose, free fatty acids (FFAs), and GLP-1 stimuli (33). With the development of T2DM, β-cell deficit and increased β-cell death occurred, resulting in an increasing β-cell mass loss (34). Dysfunction of β-cells serves as a critical factor during the development of this disease. Reduced GSIS (glucose-stimulated insulin secretion) is commonly found in symptomatic T2DM patients (35). Herein, by stimulating rats’ β-cell line INS-1 with cholesterol, we observed inhibited cell viability and promoted cell viability, suggesting the cholesterol-induced lipotoxicity on β-cells caused β-cell mass loss. By achieving miR-344-5p overexpression in cholesterol-stimulated β-cells, cholesterol-caused suppression on cell viability and decrease in GSIS were significantly reversed, and cholesterol-induced cell apoptosis and increases in intracellular cholesterol level and lip droplet deposition were inhibited. These data indicate that miR-344-5p reverses lipotoxicity-caused loss of β-cell mass and function.

Apoptosis is a morphologically and biochemically distinct form of programmed cell death that plays an essential role during embryologic development, after birth, and during adulthood (36). The intrinsic pathway can be initiated through internal signals, including Bax, Bcl-2, cytochrome C, and caspase-9/-3/-7. Moreover, Bax protein is involved in the inhibitory effect of Bcl-2 protein on apoptosis inhibition. Intrinsic apoptosis is triggered by insertion of Bax into the outer mitochondrial membrane. Formation of Bax oligomeric pores at the mitochondrial outer membranes is prerequisite for the release of cytochrome C into the cytoplasm to induce the formation of apoptosome, thus activating caspase-9, -3, -7 and resulting in cell death (36, 37). In the present study, cholesterol stimulation significantly increased pro-apoptotic Bax and cleaved-caspase 3 but decreased anti-apoptotic Bcl-2, thus inducing β-cell death and loss of β-cell mass. After overexpressing miR-344-5p in β-cells, cholesterol-induced changes in apoptosis-related factors were significantly reversed, and cholesterol-induced β-cell apoptosis was inhibited. Thus, miR-344-5p could affect apoptotic caspase 3/Bax signaling to inhibit β-cell apoptosis.

Activating IGF-1R *via* the binding of ligand induces autophosphorylation and mitogenic signaling through PI3K/Akt/mTOR and RAS/RAF/MAPK/ERK pathways (38, 39). The Ras-MAPK arm of the insulin signaling pathway serves vital roles in the proliferation and survival of pancreatic β-cells (40, 41). In the present study, cholesterol stimulation also significantly suppressed the phosphorylation of AKT, MAPK, and ERK. Similarly, miR-344-5p overexpression in β-cells reversed the phosphorylation of AKT, MAPK, and ERK, suggesting that miR-344-5p also affects the MAPK/ERK signaling to modulate β-cell functions.

Regarding the downstream mechanism, we analyzed online dataset for miR-344-5p targets, and miR-344-5p was found to directly bind to Cav1. Cav1 is one of the cholesterol-binding membrane proteins coating the intracellular surface of the small flask-shaped invagination caveolae (27, 42). Cav1 is able to interact with a number of caveolae-localized signaling molecules that contain G proteins, Src family tyrosine kinases, eNOS (endothelial nitric oxide synthase), and many ion channels through caveolin scaffolding domains in its NH2-terminal regions (43, 44). Thus, Cav1 is heavily involved in modulating cell signaling events, including cell adhesion, apoptosis, migration, and senescence (45, 46). Cav1 has exerted its effect on insulin signaling and lipid metabolism within the liver, adipose tissues, and skeletal muscles to influence diabetes development. In the present study, Cav1 could be negatively regulated by miR-344-5p. Cav1 silencing in β-cells also significantly attenuated cholesterol-induced β-cell apoptosis and dysfunction. More importantly, the functions of miR-344-5p inhibition upon β-cell apoptosis and dysfunction could be significantly reversed by Cav1 silencing.

In conclusion, the miR-344-5p/Cav1 axis modulates cholesterol-induced β-cell apoptosis and dysfunction. The apoptotic caspase 3/Bax signaling and MAPK/ERK signaling might be involved.

## Data Availability Statement

Publicly available datasets were analyzed in this study. This data can be found here: 1. GEO DataSets; Accession number: GSE57573; Link: https://www.ncbi.nlm.nih.gov/geo/query/acc.cgi?acc=GSE57573. 2. GEO DataSets; Accession number: GSE110234; Link: https://www.ncbi.nlm.nih.gov/geo/query/acc.cgi?acc=GSE110234.

## Ethics Statement

The studies involving human participants were reviewed and approved by the ethical standards of Third Xiangya Hospital. The patients/participants provided their written informed consent to participate in this study.

## Author Contributions

XS and LZ contributed to experimental design and supervising the whole experimental process. GJ and PL were involved in the experimental conducting. XS and WL contributed to the data analysis and manuscript preparation. JL and LZ revised the work critically for important intellectual content. LZ collected grants. All authors contributed to the article and approved the submitted version.

## Funding

This work was supported by the New Xiangya Talent Projects of Third Xiangya Hospital of Central South University (grant number JY201628) and the Natural Science Foundation of Hunan Province, China (2021JJ31039).

## Conflict of Interest

The authors declare that the research was conducted in the absence of any commercial or financial relationships that could be construed as a potential conflict of interest.

## Publisher’s Note

All claims expressed in this article are solely those of the authors and do not necessarily represent those of their affiliated organizations, or those of the publisher, the editors and the reviewers. Any product that may be evaluated in this article, or claim that may be made by its manufacturer, is not guaranteed or endorsed by the publisher.

## Supplementary Material

Supplementary Figure 1 Selection of miRNAs related to diabetes β-cell dysfunction. **(A)** Volcano plots of abnormally upregulated and downregulated miRNAs in dorsal root ganglia tissues between diabetic and normal rats based on GSE110234. Green plots represent downregulated miRNAs, and red plots represent upregulated miRNAs. **(B)** Hierarchical clustering heatmap showing the differentially expressed 14 miRNAs in dorsal root ganglia tissues between diabetic and normal rats based on GSE110234.

Supplementary Figure 2 The role of palmitic acid (PA) on INS-1 cells’ lipotoxicity and the function of miR-344 regulates PA-induced lipotoxicity. **(A)** INS-1 cells were treated with 0.5 mM PA for 24 h and examined for cell viability by MTT assay. **(B)** The cell apoptosis was examined by Flow cytometry assay. **(C)** The protein levels of Bcl-2, Bax, and cleaved-caspase 3 were detected by immunoblotting, and β-actin was applied as an endogenous control. **(D)** The expression level of miR-344-5p was determined by real-time PCR. **(E)** INS-1 cell was transfected with miR-344-5p mimic or inhibitor for 48 h, treated with 0.5 mM PA for 24 h, and examined for cell viability by MTT assay. **(F)** The intracellular cholesterol levels were detected by cholesterol assay kits. **(G)** The glucose-stimulated insulin secretion (GSIS) was measured by an insulin ELISA kit. **P* < 0.05, ***P * < 0.01 compared to Control group.
