# Supplementary figures and images for: miR-344-5p Modulates Cholesterol-Induced β-Cell Apoptosis and Dysfunction Through Regulating Caveolin-1 Expression
Source: Front Endocrinol (Lausanne). 2021 Jul 28;12:695164. doi: 10.3389/fendo.2021.695164 (PMC8355992; doi:10.3389/fendo.2021.695164)

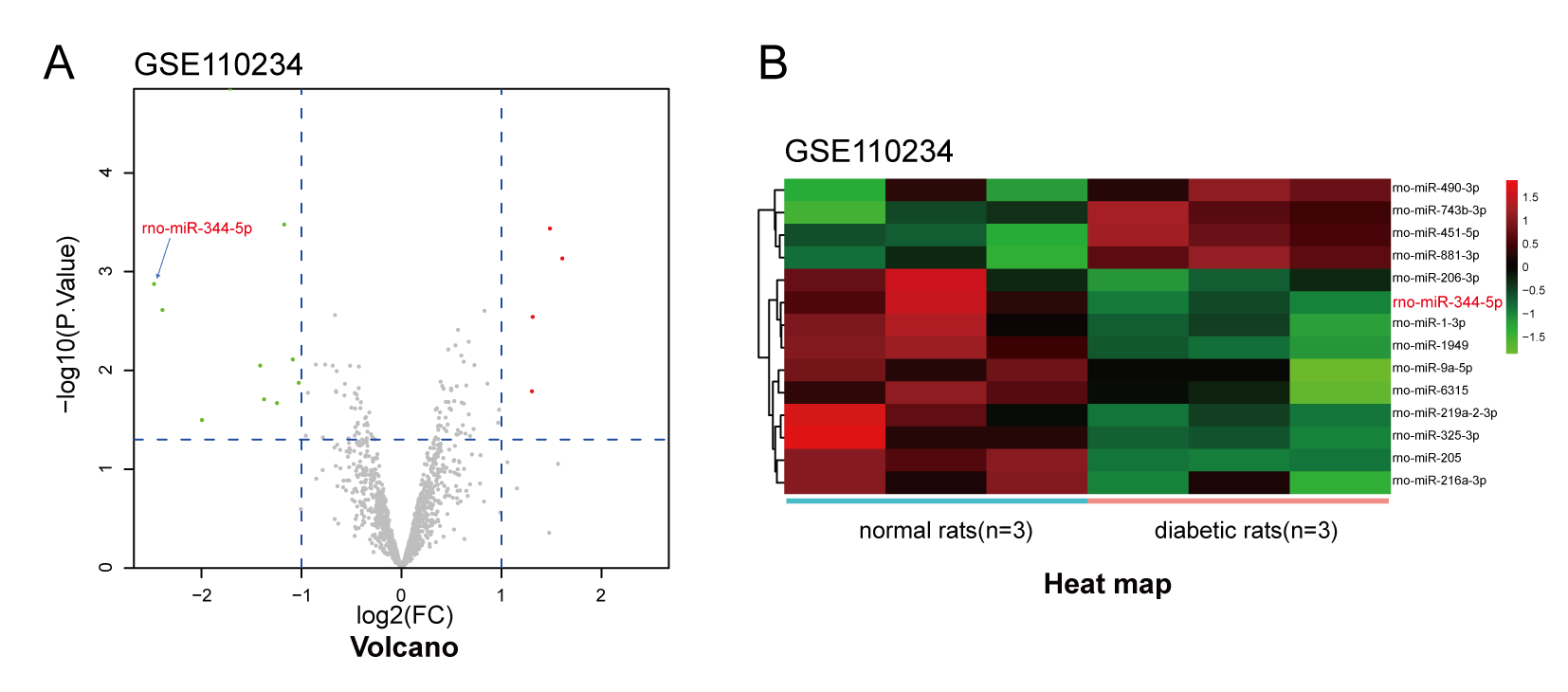

Supplement: Supplementary Figure 1 — Selection of miRNAs related to diabetes β-cell dysfunction. (A) Volcano plots of abnormally upregulated and downregulated miRNAs in dorsal root ganglia tissues between diabetic and normal rats based on GSE110234. Green plots represent downregulated miRNAs, and red plots represent upregulated miRNAs. (B) Hierarchical clustering heatmap showing the differentially expressed 14 miRNAs in dorsal root ganglia tissues between diabetic and normal rats based on GSE110234. [file Image_1.tif]

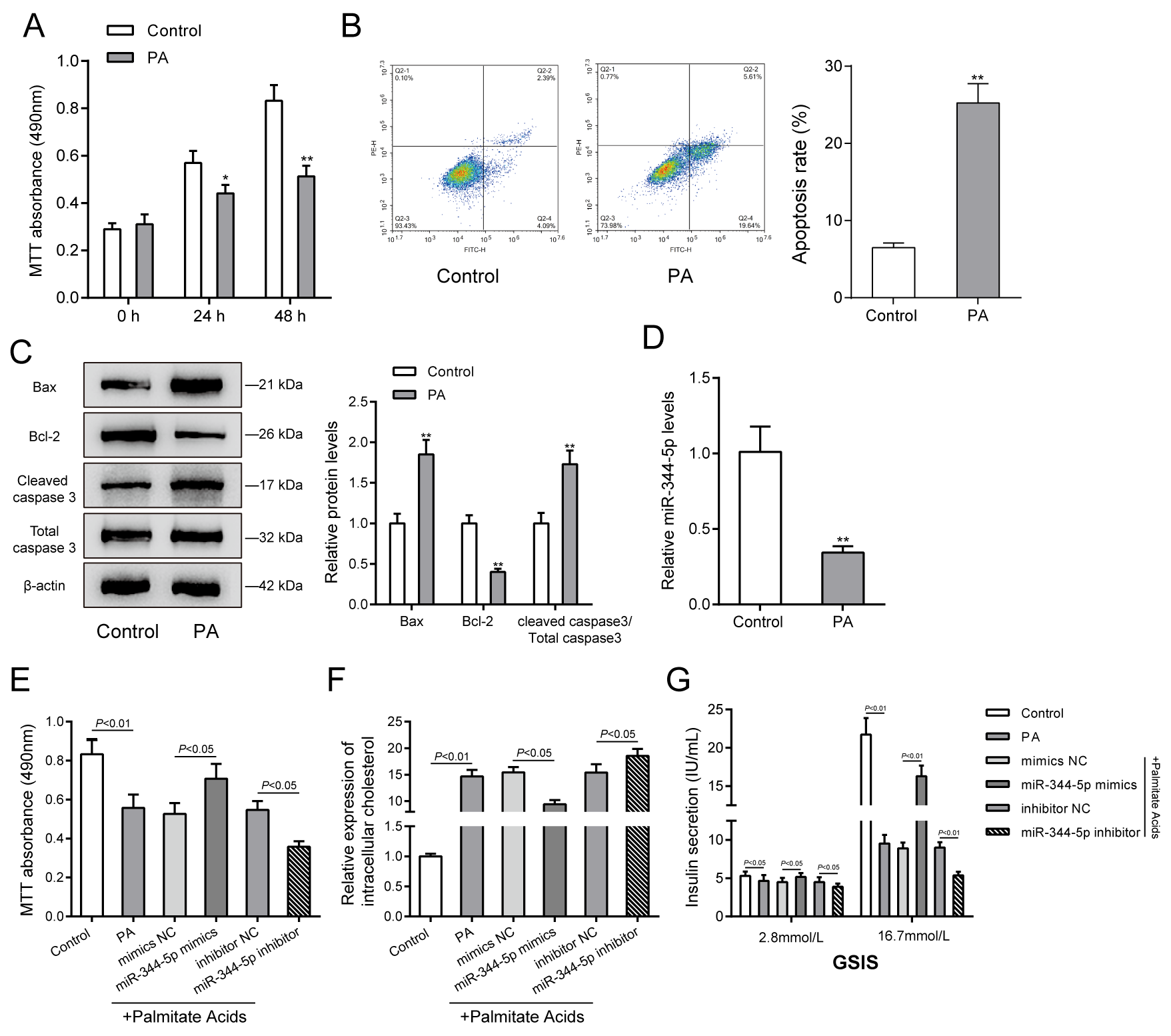

Supplement: Supplementary Figure 2 — The role of palmitic acid (PA) on INS-1 cells’ lipotoxicity and the function of miR-344 regulates PA-induced lipotoxicity. (A) INS-1 cells were treated with 0.5 mM PA for 24 h and examined for cell viability by MTT assay. (B) The cell apoptosis was examined by Flow cytometry assay. (C) The protein levels of Bcl-2, Bax, and cleaved-caspase 3 were detected by immunoblotting, and β-actin was applied as an endogenous control. (D) The expression level of miR-344-5p was determined by real-time PCR. (E) INS-1 cell was transfected with miR-344-5p mimic or inhibitor for 48 h, treated with 0.5 mM PA for 24 h, and examined for cell viability by MTT assay. (F) The intracellular cholesterol levels were detected by cholesterol assay kits. (G) The glucose-stimulated insulin secretion (GSIS) was measured by an insulin ELISA kit. *P < 0.05, **P < 0.01 compared to Control group. [file Image_2.tif]
