# Supplementary material for: miR-344-5p Modulates Cholesterol-Induced β-Cell Apoptosis and Dysfunction Through Regulating Caveolin-1 Expression
Source: Front Endocrinol (Lausanne). 2021 Jul 28;12:695164. doi: 10.3389/fendo.2021.695164 (PMC8355992; doi:10.3389/fendo.2021.695164)
Supplement: Supplementary file 3 [file Table_1.docx]

**Table S1** **Differentially expressed miRNAs between diabetic and normal rats based on GSE110234**

| **genesymbol** | **logFC** | **AveExpr** | **P.Value** |
| --- | --- | --- | --- |
| rno-miR-344-5p | -2.477150583 | 3.539697097 | 0.001339297 |
| rno-miR-1-3p | -2.393319889 | 3.331804056 | 0.002452768 |
| rno-miR-9a-5p | -1.997440655 | 3.340925728 | 0.031881472 |
| rno-miR-205 | -1.714877116 | 5.707332331 | 1.41E-05 |
| rno-miR-219a-2-3p | -1.415602801 | 1.110571643 | 0.008948438 |
| rno-miR-6315 | -1.376601339 | 2.164402715 | 0.019663056 |
| rno-miR-206-3p | -1.247631394 | 6.504342303 | 0.021467501 |
| rno-miR-1949 | -1.175437611 | 6.950980917 | 0.00033444 |
| rno-miR-325-3p | -1.089441333 | 2.284495611 | 0.007765694 |
| rno-miR-216a-3p | -1.029998328 | 1.831829114 | 0.013378962 |
| rno-miR-490-3p | 1.302609093 | 2.446123343 | 0.016309269 |
| rno-miR-743b-3p | 1.31014094 | 2.731375914 | 0.002878842 |
| rno-miR-451-5p | 1.482599731 | 2.917591856 | 0.000366898 |
| rno-miR-881-3p | 1.606427593 | 3.366519519 | 0.000737515 |
