# Supplementary material for: miR-344-5p Modulates Cholesterol-Induced β-Cell Apoptosis and Dysfunction Through Regulating Caveolin-1 Expression
Source: Front Endocrinol (Lausanne). 2021 Jul 28;12:695164. doi: 10.3389/fendo.2021.695164 (PMC8355992; doi:10.3389/fendo.2021.695164)
Supplement: Supplementary file 4 [file Table_2.docx]

**Table S2. The sequence of siRNA Cav1 and miR-344-5p mimics and inhibitor**

| Gene | Sequence |
| --- | --- |
| siRNA1 | 5'-ACGCGCACACCAAGGAGATTGATCT-3' |
| siNC-1 | 5'-ACGACACAACCGAGGGTTAAGCTCT-3' |
| siRNA2 | 5'-GGCATCTACTTTGCCATCCTCTCTT-3' |
| siNC-2 | 5'-GGCTCCATTGTCCTACCCTTTACTT-3' |
| mimics NC | 5’-UUCUCCGAACGUGUCACGUTT-3’ |
| miR-344-5p mimics | 5'-AGUCAGGCUCCUGGCAGGAGUC-3' |
| inhibitor NC | 5'-CAGUACUUUUGUGUAGUACAA-3' |
| miR-344-5p inhibitor | 5’-GACUCCUGCCAGGAGCCUGACU-3' |
