# Supplementary material for: miR-344-5p Modulates Cholesterol-Induced β-Cell Apoptosis and Dysfunction Through Regulating Caveolin-1 Expression
Source: Front Endocrinol (Lausanne). 2021 Jul 28;12:695164. doi: 10.3389/fendo.2021.695164 (PMC8355992; doi:10.3389/fendo.2021.695164)
Supplement: Supplementary file 5 [file Table_3.docx]

**Table S3. The primer sequence for qPCR assay**

| **Gene** | **Forward** | **Reverse** |
| --- | --- | --- |
| Cav1 | CCCAAGCATCTCAACGACGAC | GATGGTAGACAGCAAGCGGTAAAA |
| Ngfr | GCTGATGCTGAATGCGAAGAGA | AGCAGCCAAGATGGAGCAATAG |
| Plag1 | AACAACTTTACCTTTGGGAATGACG | TGAAAGCAAGATGATGAGAGGGG |
| Papss2 | AAGGTGGGGACTGGCTTGTTGGT | CCGTTGTGGACAGGATTGCGTAA |
| Itpkb | CTCTGGACCCCAACTCAGCCTTTT | CCTCAGCACATCCGCCATTAACC |
| Ret | ATTCCCGTCAAATGGATGGC | ATCTCCCATAGCAGCACTCCAA |
| miR-344-5p | AGTCAGGCTCCTGGCA | CAGTGCGTGTCGTGGA |
| GAPDH | GACATCAAGAAGGTGGTGAAGC | TGTCATTGAGAGCAATGCCAGC |
| U6 | CTCGCTTCGGCAGCACA | AACGCTTCACGAATTTGCGT |
