# Supplementary material for: miR-344-5p Modulates Cholesterol-Induced β-Cell Apoptosis and Dysfunction Through Regulating Caveolin-1 Expression
Source: Front Endocrinol (Lausanne). 2021 Jul 28;12:695164. doi: 10.3389/fendo.2021.695164 (PMC8355992; doi:10.3389/fendo.2021.695164)
Supplement: Supplementary file 6 [file Table_4.docx]

**Table S4 Differentially expressed mRNAs between normal and hIAPP-treated INS-1E cells based on GSE57573**

| **genesymbol** | **logFC** | **AveExpr** | **adj.P.Val** |
| --- | --- | --- | --- |
| Crispld1 | 2.913784 | 9.553746 | 0.00421 |
| Tnfrsf11b | 2.684271 | 9.534962 | 0.007015 |
| Insrr | 2.199303 | 7.56826 | 0.006527 |
| Itpkb | 2.099189 | 8.732096 | 0.005694 |
| Tmem176a | 2.048472 | 8.51774 | 0.010076 |
| Ngfr | 1.985486 | 9.357737 | 0.009195 |
| Derl3 | 1.838193 | 8.973016 | 0.006527 |
| Car8 | 1.749605 | 11.81028 | 0.006722 |
| Slc22a1 | 1.624776 | 8.782685 | 0.013289 |
| Slc16a12 | 1.554099 | 10.42329 | 0.007015 |
| Vstm2b | 1.465026 | 9.893467 | 0.007015 |
| Galnt14 | 1.445193 | 11.01326 | 0.006722 |
| Rasgef1c | 1.385825 | 9.332384 | 0.007443 |
| Sdf2l1 | 1.36411 | 9.795565 | 0.009629 |
| Ppp1r14c | 1.343414 | 8.390868 | 0.008955 |
| Papss2 | 1.331161 | 9.292184 | 0.007015 |
| Igf1 | 1.293038 | 11.21499 | 0.007443 |
| Hsd17b11 | 1.281942 | 9.015636 | 0.012032 |
| Nqo1 | 1.271054 | 8.308131 | 0.02518 |
| Cav1 | 1.262377 | 7.033168 | 0.029964 |
| Tff3 | 1.238723 | 10.80676 | 0.010076 |
| Klk1 | 1.225996 | 10.11595 | 0.009056 |
| Slc26a4 | 1.224335 | 8.231542 | 0.017185 |
| Loxl2 | 1.18031 | 8.832649 | 0.010076 |
| Trpv1 | 1.172156 | 10.12392 | 0.008232 |
| Ppap2b | 1.13669 | 7.718592 | 0.010076 |
| Plag1 | 1.132412 | 8.163278 | 0.023212 |
| Ret | 1.103455 | 10.83091 | 0.009629 |
| Epha5 | 1.10103 | 8.514231 | 0.010076 |
| Arap2 | 1.088013 | 10.00403 | 0.014384 |
| Rai2 | 1.077213 | 10.82353 | 0.010396 |
| Slc17a9 | 1.051399 | 9.076958 | 0.010314 |
| LOC689926 | 1.00836 | 9.068868 | 0.01445 |
